# The feasibility and acceptability of collaborative learning in improving health worker performance on adolescent health: findings from implementation research in Moldova

**DOI:** 10.1186/s12913-019-4158-2

**Published:** 2019-05-28

**Authors:** Galina Lesco, Frances Squires, Viorel Babii, Nadejda Bordian, Olga Cernetchi, Adriane Martin Hilber, Venkatraman Chandra-Mouli

**Affiliations:** 1 National Resource Centre in Youth Friendly Health Services (NEOVITA) and National Coordinator of Healthy Generation Project, Health for Youth Association, 19 Socoleni Str, MD 2020 Chisinau, Republic of Moldova; 2 Swiss Centre for International Health, Swiss Tropical and Public Health Institute, Socinstrasse 57, P.O. Box, CH-4002 Basel, Switzerland; 3 Childhood Studies and Children’s Rights, Healthy Generation Project, Health for Youth Association, 19 Socoleni Str, MD 2020 Chisinau, Republic of Moldova; 4 YFHS Monitoring and Evaluation, National Resource Centre in Youth Friendly Health Services (NEOVITA), 19 Socoleni Str, MD 2020 Chisinau, Republic of Moldova; 5 Department of Obstetrics and Gynecology, Nicolae Testemitanu State University of Medicine and Pharmacy of the Republic of Moldova, 165, Stefan cel Mare si Sfant Bd, MD-2004 Chisinau, Republic of Moldova; 6 Department of Reproductive Health and Research, World Health Organisation, Avenue Appia 20, 1202 Genève, Switzerland

**Keywords:** Adolescent, Adolescent health services, Collaborative learning, Health worker performance, Moldova, Reproductive health services, Youth, Youth friendly

## Abstract

**Background:**

Collaborative learning has been shown to be effective in improving health worker performance, but relatively little is known about the feasibility or acceptability of collaborative learning in youth-friendly health services (YFHS). This paper describes the characteristics, feasibility and acceptability of a collaborative learning approach implemented in YFHS in Moldova as part of a national scaling up process.

**Methods:**

We gathered and analysed data on the number, location, themes, and participants of sessions, as well as benefits and challenges of collaborative learning, using two information sources: 1) formal reports on collaborative learning sessions, and 2) two questionnaires conducted with participants and moderators.

**Results:**

Collaborative learning sessions have been implemented in 30 out of 35 YFHS in Moldova. In 2016, 464 collaborative learning sessions were conducted. Sessions were conducted one to three times per month, had a mean of 15 participants and an average duration of two - three hours. 74.3% of participants (*n* = 6942) were from rural areas and 55.1% were health professionals. The most common topics in 2016 were adolescent health and YFHS (159 of 464 sessions), sexual and reproductive health (103 sessions), and violence (76 sessions).

Reported benefits for participants of collaborative learning fell into three categories: 1) improved knowledge on adolescent health / development and use of evidence-based resources; 2) strengthened teamwork and cooperation; and 3) empowerment to provide high quality, youth-friendly care. Moderators identified benefits for the quality, youth-friendliness, and positioning of YFHS as centres of excellence on adolescent health. Challenges included the time and resources required to start and maintain the program, developing a constructive multi-disciplinary learning culture, and ensuring the involvement of stakeholders from outside YFHS.

**Conclusion:**

This study confirms that collaborative learning within YFHS is feasible and acceptable, and offers benefits to both participants and YFHS. Collaborative learning may be a valuable strategy to improve the quality and youth-friendliness of services. It may also be relevant to key challenges in scaling up YFHS such as increasing utilisation and achieving long-term sustainability. Further research is required to confirm our results in other settings and to examine the effects of collaborative learning at the outcome and impact level.

## Background

Collaborative learning has been increasingly implemented in healthcare settings in recent years. Defined as an approach to teaching and learning in which groups of learners work together, collaborative learning typically involves group discussions, with participants cooperating to solve a problem, complete a task, or otherwise advance learning [1]. This approach appears to be effective in improving the performance of health workers, with a large systematic review conducted in low-resource settings showing greater effect from group processes, especially when combined with other approaches such as good quality training [2]. This finding was confirmed by a recent Lancet Global Health Commission on high quality health systems, which noted that, “District-led collaborative learning has the potential to foster improved quality through better system functioning and communication” [3].

Relatively little is known about the feasibility or effectiveness of collaborative learning to improve health worker performance in youth-friendly health services (YFHS), which aim to prevent, detect and treat health conditions among adolescents and youth by delivering health services in ways that are accessible and acceptable to young people themselves (i.e. they are able and willing to obtain them). Several countries have systematically scaled up YFHS in recent years, but consistent delivery of high quality services remains a key challenge [4].

**Fig. 1:**
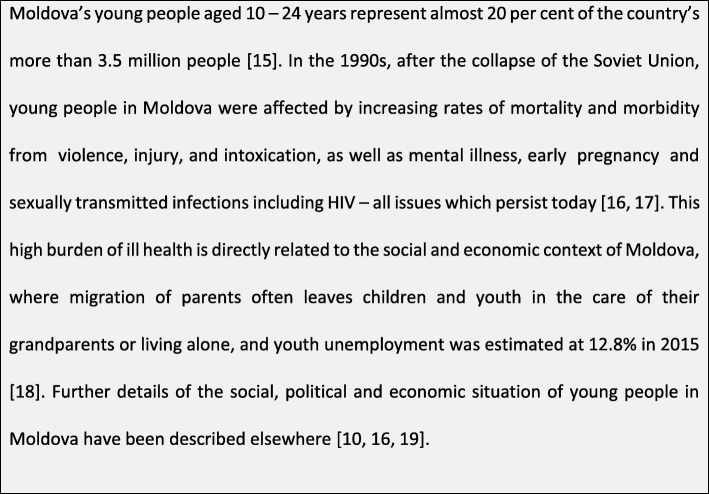
The situation of young people in Moldova

**Fig. 2:**
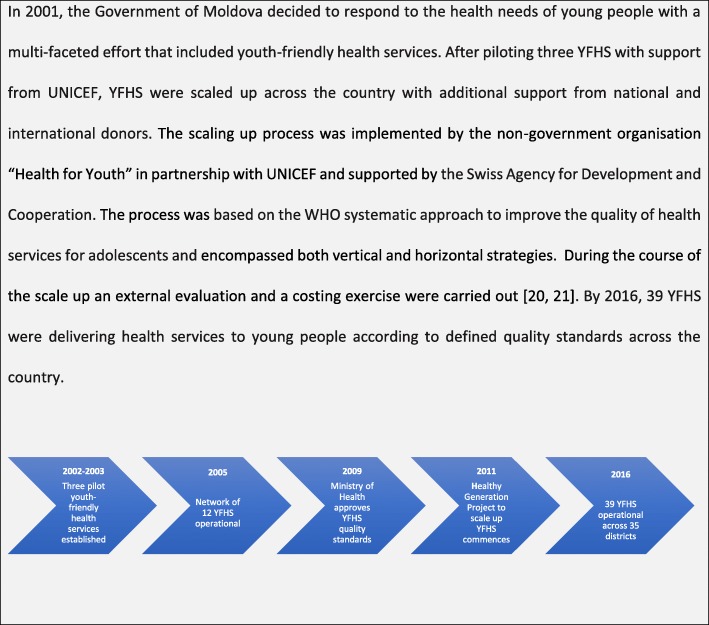
Scaling up process for youth-friendly health services in the Republic of Moldova

This paper explores whether collaborative learning in YFHS is feasible and acceptable as a means for improving YFHS quality by examining recent experience in the Republic of Moldova, an ex-Soviet country in Eastern Europe, which has implemented collaborative learning across 39 YFHS as part of a national scale up of youth-friendly health services since 2012 (see Figs. 1 and 2) [5–12]. We aimed to describe the characteristics of the collaborative learning approach implemented in Moldova, including the number of districts and YFHS involved, number of sessions, profile of participants and key themes of sessions. We also explored the key benefits and challenges of the collaborative learning approach for participants and YFHS.

## Methods

### Data collection

We collected data from two sources: 1) internal reporting on collaborative learning sessions and 2) two questionnaires conducted with participants and moderators of collaborative learning sessions.

To investigate the characteristics of collaborative learning approach in Moldova and its feasibility, we examined regular internal reporting on all collaborative learning sessions conducted in YFHS across the country in 2016. This reporting was required under the Healthy Generation Project and provided detailed information on the characteristics of the sessions (number, location and duration of sessions; topics covered; number, gender and profession of the participants.) On the basis of these reports, YFHS managers (who are usually one of the local moderators) collated three-monthly reports on collaborative learning sessions, which were presented and discussed at national-level collaborative learning sessions. We also collected national-level reports, which were produced half-yearly by the National Resource Centre attached to the Neovita youth-friendly health service in the capital city Chisinau for reporting to the Ministry of Health and donors.

In order to assess the acceptability of the collaborative learning approach to participants and facilitators and to explore their perceptions about the benefits and challenges of the approach, we conducted two qualitative surveys. Purposeful sampling was used to administer the surveys, completion of which was entirely voluntary. The questionnaires were developed in consultation with the World Health Organisation (WHO), with the primary aim of collecting feedback to refine the implementation of collaborative learning across the country.

The first survey, which was conducted in May 2015, asked participants and facilitators about the perceived purpose, benefits and challenges of the sessions. The questionnaire additionally asked YFHS managers for a description of sessions at their centre as well as the profile of participants. All YFHS managers who had been implementing collaborative learning sessions for more than two years completed the questionnaire. At the first CLS held in May 2015 in each YFHS, two moderators and four participants were asked to complete the questionnaire. Where more than four participants wished to complete the survey, the first four to volunteer were selected. A total of 16 moderators and 32 participants from eight YFHS completed the questionnaire.

The second questionnaire was conducted with 20 session moderators (from 20 different YFHCs) at a national-level quarterly CLS in April 2016. This questionnaire asked moderators to describe the benefits of CLS for participants and for the youth-friendly health services, to identify challenges in conducting CLS and to make proposals for improvement of YFHS.

### Data analysis

**Fig. 3:**
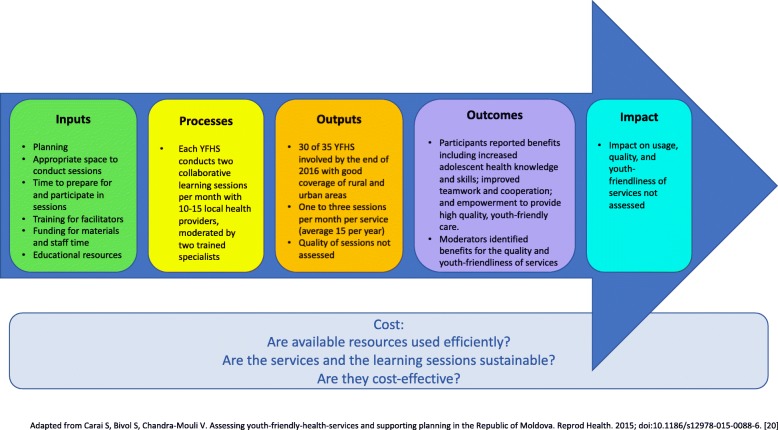
Evaluation framework for assessing collaborative learning

In the data analysis phase, we set out to synthesise learnings from the Moldovan experience at the input and output level (see Fig. 3). Outcomes reported by participants and moderators were also collected, but not independently assessed. The effect of the approach on usage, quality or youth-friendliness of services (impact level) is beyond the scope of this study.

To analyse results of the surveys and reports we used a combination of quantitative and qualitative methods. Quantitative methods were used to evaluate frequency distributions of CLS by district, location, topics discussed and the profile and number of participants. For qualitative information gathered, such as responses to open-ended questions, themes were identified and based on this, conclusions reached on issues such as how moderators and participants perceived CLS.

## Results

### Contextual background: collaborative learning in youth-friendly health services in Moldova

The national scaling up process for YFHS in Moldova implemented training of healthcare providers to deliver high quality services to young people. In 2011–2012 a comprehensive system of in-service training was developed in accordance with the WHO Adolescent Job Aid [13] and Orientation Program on Adolescent Health for Health-care Providers [14], as well as the EuTEACH training program [15] and the national quality standards for youth-friendly health services. This training was implemented in all districts of Moldova between 2012 and 2016, varying from three days of basic training in youth-friendly healthcare for family doctors and nurses, to 10 days of extended training for specialist doctors from YFHS. A three-day learning program on adolescent health promotion was also developed for community and school workers (e.g. school nurses, psychologists, social workers and teachers).

The scaling up process also incorporated collaborative learning processes, pre-empting the WHO Global Standards for Quality Healthcare for Adolescents, which recommend the implementation of a supportive, non-punitive system for supervising adolescent healthcare providers with an emphasis on problem-solving approaches [16]. The first collaborative learning meetings were conducted at the end of 2012 by four YFHS, with 30 (of 35) YFHS applying the approach by the end of 2016. Each CLS aims to improve the knowledge, understanding and motivation of health professionals working in adolescent healthcare and health promotion to ensure delivery of high quality health services.

Collaborative learning sessions are conducted according to national YFHS regulations, which stipulate that each YFHS should conduct two CLS per month. Each CLS brings together 10–15 local health providers (general practitioners, specialists and nurses) and/or school and community workers from the local YFHS, other health services, and schools for around three hours and focuses on a subject related to one of the seven priority adolescent health problems described in the YFHS standards (see Tables 1 and 2). The subject of each session is generally selected in consultation with participants with consideration of the local epidemiological situation (for example, increased incidence of violence in a particular area could prompt a session on this issue). The sessions are moderated by two specialists from the YFHS (often also YFHS managers), who facilitate discussions, offer teaching and document the session. National-level collaborative learning sessions are also conducted quarterly, where YFHS from different districts report on their results and share experiences and challenges.

**Table 1:** The seven priority adolescent health problems addressed by YFHS in Moldova

1. Sexually transmitted infections and HIV	
2. Early pregnancy	
3. Pubertal disorders	
4. Nutritional problems and eating disorders	
5. Psychological wellbeing, including mental health problems, depression and suicide	
6. Substance abuse and misuse	
7. Health problems related to violence and human trafficking

**Table 2:** Schedule of a typical collaborative learning session

Introduction (30–40 min) Welcome and introductions, discussion of changes in practice after basic training in adolescent health care/promotion or previous collaborative learning session	
Sharing of experience (60 mins) Discussion of existing and best practices on the specific topic of the session	
Teaching (30 mins) Teaching on a selected issue, moderated by a YFHC specialist (based on WHO Adolescent Job Aid, current evidence, or other relevant resources)	
Planning (30–40 min) Discussion to identify solutions and actions to improve practice to address the discussed health issue or problem; updating and sharing of the district’s referral framework for adolescent health.	

### Characteristics and feasibility of the collaborative learning approach

Our results showed that the collaborative learning approach was implemented in 30 of 35 districts in Moldova, with a total of 31 YFHS participating. A total of 464 collaborative learning sessions were held in 2016. YFHS conducted learning sessions from one to three times per month, giving an average of almost 15 (14.96) sessions per service per year, or 1.25 sessions per month at each service. The duration of each session was two to three hours.

A total of 6942 health providers and community workers participated in the sessions (with an average of just under 15 participants per session). Both health professionals and community workers such as teachers and school counsellors well represented. Over half (50.9%) of sessions were conducted only with healthcare providers, with the remaining sessions fairly evenly split between groups of community and school workers and other stakeholders (26.7%), and mixed groups (22.4%). Nearly 80% of provider participants became involved in CLS after completing the basic training in adolescent healthcare or promotion. It was noted that the vast majority of participants were female (83.3%, *n* = 5780), reflecting the high proportion of women working in the health, teaching, and community work professions.

The majority (70%, *n* = 325) of CLS took place in town-based YFHCs. The sessions nonetheless reached a significant number of rural participants (74.3% of participants), which offered the opportunity for community and school workers to improve their understanding of the YFHC model. The other 30% of sessions were conducted in rural areas that do not have ready access to town-based YFHCs, with the aim of facilitating the participation of local communities (see Table 3).

**Table 3:** Location, number, and participant profile of collaborative learning sessions, all YFHCs, 2016

Location	Number of sessions	Total number of participants	Number of health provider participants	Number of community worker participants	Percentage of participants from rural areas
Chisinau	4	148	140	8	0
Anenii Noi	20	322	228	94	93.2%
Balti	24	253	30	223	59.3%
Briceni	8	94	62	32	74.5%
Cahul	22	244	173	71	73.4%
Calarași	7	66	45	21	24.2%
Cantemir	6	73	45	28	68.5%
Ceadîr-Lunga	9	230	80	150	87%
Cimișlia	20	333	169	164	77.8%
Comrat	8	123	30	93	81.3%
Criuleni	21	189	142	47	68.8%
Dondușeni	13	134	85	49	67.1%
Drochia	24	371	251	120	80.9%
Edinet	24	502	301	201	84.9%
Falesti	21	230	202	28	73.9%
Floresti	24	606	240	366	70.3%
Glodeni	11	119	95	24	71.4%
Leova	24	505	204	301	73.3%
Nisporeni	17	166	124	42	80.1%
Ocnita	16	167	121	46	59.3%
Orhei	19	186	72	114	67.2%
Riscani	16	172	147	25	72.7%
Singerei	23	368	215	153	76.1%
Soldanesti	1	30	0	30	100%
Soroca	10	90	43	47	66.7%
Stefan Voda	15	225	42	183	78.7%
Straseni	17	179	120	59	56.4%
Taraclia	2	31	13	18	100%
Telenesti	8	140	88	52	60%
Ungheni	22	465	289	176	66.7%
Vulcanesti	8	181	32	149	61.9%
Total	464	6942	3828 (55.1%)	3114 (44.9%)	74.3%

The most common topics for sessions in 2016 were adolescent health and YFHS (159 of 464 sessions), sexual and reproductive health (103 sessions), and violence (76 sessions). Table 4 presents a breakdown of CLS themes according to participant type. For community workers, the bio-psycho-social development of adolescents was the most common topic, whereas health providers focussed on more sensitive topics such as prevention of STIs, unwanted pregnancy and unsafe abortion.

**Table 4:** CLS themes and participants, 2016

Theme of session	Total number of sessions	Number of sessions with health provider participants	Number of sessions with community worker participants	Number of sessions with mixed groups
Adolescent health and YFHS				
Bio-psycho-social development of adolescents	53	19	24	10
Puberty development and disorders; psycho-sexual development	27	15	7	5
Orientation to YFHS and improving inter-sectorial cooperation	23	14	3	6
Health promotion	26	10	7	9
Importance of adolescent health for society	16	9	5	2
The adolescent-parent relationship and conflicts	6	3	3	0
The family’s role in promoting adolescent health	6	2	2	2
Volunteer roles within YFHCs	1	1	0	0
Education and career considerations for adolescents	1	0	1	0
Sexual and reproductive health				
Prevention of STIs/HIV, unwanted pregnancy and unsafe abortion	86	54	15	17
Sexuality education of adolescents	17	8	5	4
Violence				
Family violence and violent behaviour among adolescents	50	31	8	11
Bullying in schools	19	5	8	6
Prevention of human trafficking of young people	7	3	3	1
Mental health				
Adolescents’ emotional wellbeing and psychological development	11	5	0	6
Depression and suicide prevention among adolescents	9	2	3	4
Risk behaviours among adolescents; prevention of substance abuse	28	10	13	5
Prevention of mental health problems among adolescents	10	4	5	1
General health				
Nutritional disorders in adolescence, including obesity and undernutrition	18	10	3	5
Acne and other skin problems in adolescents	4	2	1	1
Cervical cancer prevention	4	2	1	1
Trauma and injuries in adolescents	2	1	1	0
Breast development and pathologies	1	1	0	0
Tuberculosis prevention in young people	1	1	0	0
Counselling and communication with adolescents				
HEADS assessment and using the WHO Job Aid	10	10	0	0
Individual counselling of adolescents	6	4	2	0
Effective communication and healthy inter-personal relationships	5	2	2	1
Prevention of professional burn-out syndrome	6	2	1	1
Marginalised and vulnerable groups				
Adolescents at risk; concepts of vulnerability	12	6	1	5
Integration of adolescents with special needs at school	1	0	0	1

Each CLS identified ways to improve adolescent health practice. The most common intended activities were:

- Strengthening of referral frameworks;
- Creation of new partnerships (e.g. with local non-governmental organisations, social services, police); and
- Planning of new activities with partners (e.g. information sessions for parents and/or adolescents, advocacy meetings with local authorities, or other health promotion activities).

### Acceptability and perceived benefits of collaborative learning

The two questionnaires provided information on the purpose and benefits of collaborative learning, as perceived by participants and facilitators. Participants had three main interpretations of the purpose of collaborative learning:

1. To increase knowledge on a specific adolescent health subject;
2. To share good practices and successful adolescent health care models; and
3. To engage in group problem-solving.

Perceived benefits from participation in the sessions also fell into three main categories, which mirrored the perceived purpose of the sessions:

1. Increased adolescent health knowledge and skills;
2. Improved teamwork and cooperation; and
3. Empowerment to provide high quality, youth-friendly care.

The first benefit relates to obtaining new knowledge and skills related to adolescent health and development, for example by learning how to make better use of guidelines and obtain more information and education materials for their work. Specialists stated, “I became aware of available services for adolescents” (teacher, Calarasi district) and, “I learned to apply the local referral framework” (school nurse, Soroca district).

The second area centred on improving communication with colleagues and developing cooperation with other specialists and sectors. One specialist reported, “I satisfied my need to communicate with colleagues” (family doctor, Edinet district).

The third area of perceived benefit related to empowerment to “make a difference” to young people. Participants felt enabled to provide more youth-friendly care to young people, especially in sensitive areas: “After participating in CLS … I feel more confident to discuss issues related to sexuality with adolescents” (biology teacher, Cimislia district). One nurse commented, “I better understood the particularities of adolescence, and how to deal with adolescents in my practice and in my family” (family nurse, Criuleni district). A family doctor concluded, “Respectful attitudes and confidential care can improve the results of our work with adolescents a great deal” (family doctor, Edinet district).

CLS moderators had a more refined understanding of this form of collaborative learning, describing each CLS as a meeting of professionals with a common goal, i.e. learning how to practice more effectively and efficiently through the exchange of experience, and identifying challenges and solutions to them. Moderators confirmed the benefits of CLS articulated by participants, especially with regard to benefits for teamwork and cooperation. Specifically, moderators mentioned that collaborative learning offers participants the possibility to “improve cooperation” (moderator, doctor, Cimislia district) and “referral to different services” (moderator, psychologist, Edinet district), including via “creation of a functional inter-disciplinary team at the local level” (moderator, social worker, Balti district).

Moderators additionally identified benefits for the quality and youth-friendliness of services. For example, moderators reported that CLS helped participants “to have a more youth-friendly attitude and better respect confidentiality” (moderator, doctor, Soroca district), “to have a more understanding and supportive attitude to some sensitive issues such as sexuality or violence” (moderator, doctor, Falesti district), “to develop their interpersonal communication abilities,” (moderator, psychologist, Orhei district), and to improve use of existing guidelines.

YFHS managers also evoked a series of benefits to youth-friendly health services from collaborative learning sessions, including:

- Improved cooperation within the health sector and across other sectors in the area of adolescent health;
- Strengthened positioning of YFHS as trusted resource centres for adolescent health and development;
- Increased number of YFHS beneficiaries; and
- More efficient health services for adolescents, especially vulnerable groups.

This was supported by CLS reports, in which participants reported informing over 65,700 adolescents (representing almost 15% of adolescents in the country) about YFHS (see Table 5). While this is not directly attributable to involvement in the collaborative learning approach, participants also reported referring almost 11,000 young people to YFHCs, suggesting a high level of confidence in the YFHC model as an important resource for young people.

**Table 5:** Number of young people informed of or referred to YFHCs by CLS participants, 2016

Total number of CLS participants	6942
Total number of young people informed about YFHCs by CLS participants	65,783
Total number of young people referred to YFHCs by CLS participants	10,997

### Challenges in implementing collaborative learning sessions

CLS participants and moderators were generally positive about the collaborative learning approach, but identified some challenges in implementing the sessions, most of which related to the novelty of the approach and the resources (e.g. staff time and travel costs) required to run sessions.

While youth-friendly health services have been in place for some time, the application of collaborative learning is new in Moldova. In particular, learning in multi-disciplinary groups, where participants have different backgrounds and understanding of adolescent health, was a relatively new concept for many moderators and participants. One moderator noted, “Initially we didn’t know how it would work better - to have CLS only for doctors, only for nurses, only for teachers or to combine them” (psychologist, Anenii Noi district). The involvement of stakeholders from outside YFHCs, such as family doctors and school resource persons, was in fact identified as both a key strength and a challenge of the approach, especially in rural areas where health providers are often under significant time pressure in their work. “CLS with participation of family doctors and community resource persons made it possible to establish better cooperation between different actors in the local area … but it was very difficult to find a convenient time for all of them to meet” (moderator, doctor, Falesti district).

These challenges and the newness of the approach meant that significant time, energy and skill was required to start sessions and to establish a learning environment where it was possible for all participants to contribute equally. One moderator said, “CLS needs time for preparation, travel if it is organised outside of the YFHC, and effort to invite participants, especially from other sectors” (doctor, Cimislia district). Further, as noted by one of the authors who led this process (GL), the lack of normative guidance on how to conduct CLS and of functional systems to involve different sectors hindered work.

## Discussion

The learning programme implemented in YFHS in Moldova is the first time collaborative learning methods have been used as a central component of a YFHS scaling up process. Our results confirmed that this approach is feasible, with the vast majority of YFHS successfully implementing CLS across Moldova. Our results also suggest that collaborative learning in YFHS is highly acceptable to both participants and moderators and appears to have benefits for both participants and the health services.

Key benefits of collaborative learning for session participants appear to include improved knowledge and use of evidence-based resources on adolescent health; strengthened teamwork and cooperation; and increased confidence to provide high quality, holistic care, including on sensitive topics such as sexuality. Collaborative learning appears to contribute to the commitment, confidence and ability of providers to deliver youth-friendly health services in accordance with quality standards and guidelines. The regular inclusion of themes such as family planning, violence and abortion is likely to have contributed to reported benefits around improved youth-friendly attitudes of providers, including on sensitive issues, and greater awareness and observation of confidentiality. Given that confidentiality and respect are consistently identified as two of the most important aspects of youth-friendly services by young people themselves, these findings suggest collaborative learning may be a valuable tool for ensuring that YFHS deliver truly youth-friendly care [17].

Collaborative learning also appears to have benefits for youth-friendly health services, including strengthening their position as trusted resource centres for adolescents and youth, improving cooperation on adolescent health, and increasing the number of beneficiaries. Indeed, collaborative learning participants reported high numbers of referrals to YFHCs. While our study could not verify that the collaborative learning approach resulted in increased numbers of referrals to YFHCs or usage of the services by young people, it is plausible that the involvement of key stakeholders from outside YFHCs such as teachers, school counsellors and religious leaders may have resulted in increased referrals and therefore increased usage. It is also likely that improvements in youth-friendliness and acceptability of care as a result of collaborative learning will have flow-on effects in terms of service usage, and in the longer term, sustainability of services. These findings are highly relevant given well-documented challenges in ensuring the quality, youth-friendliness, usage and sustainability of youth-friendly health services during national scale up processes [5, 18, 19].

Our results suggest that the collaborative learning approach may have system-wide benefits which are highly relevant to the Moldovan context. In a highly hierarchical and traditionally heavily hospital-based health system, learning in multi-disciplinary groups was a relatively new concept for many moderators and participants but appeared to be a key contributor to benefits around teamwork, inter-sectoral cooperation, and referral pathways. Improvements in provider knowledge and confidence to provide youth-friendly care are also likely to have a positive impact on job satisfaction – potentially a crucial factor in addressing high staff turnover and net emigration of trained health providers, and thus quality of health services [20, 21]. Ongoing challenges to the sustainability of collaborative learning in YFHS are likely to be posed by the lack of regulations mandating continued professional development of health providers, and insufficient dedicated funding for YFHCs to run collaborative learning sessions.

This study is the first to examine collaborative learning as part of a national scaling up process for YFHS and adds to existing evidence on the effectiveness of collaborative learning in other health settings. However, study limitations include that we were not able to assess whether collaborative learning directly resulted in higher quality, including youth-friendliness of services, increased referrals or usage, or the long-term sustainability of services. The question of cost was also beyond the scope of this study. Our findings may furthermore have been subject to selection bias, since questionnaire participants volunteered for the task and could be more likely to have strong feelings about collaborative learning (positive or negative).

Our results provide preliminary evidence for a range of potential benefits provided by collaborative learning, and confirm that such an approach is both feasible and acceptable when implemented as part of a national YFHS scale up process. On the basis of our results and existing evidence for the benefits of collaborative learning, similar approaches could be confidently adopted by YFHS in other settings. This study provides sufficient evidence to warrant further research in Moldova and elsewhere to examine whether collaborative learning fulfils its potential at the impact level – that is, by contributing to improved quality of care, greater youth-friendliness, increased referrals to and usage of services, and long-term sustainability.

## Conclusion

Our results suggest that collaborative learning, when systematically implemented as part of a national scale-up process for YFHS, is both feasible and acceptable. The approach offers promising benefits to participants and YFHS that are likely to be highly pertinent to addressing some of the key challenges in successfully scaling up YFHS, namely ensuring quality of care and youth-friendliness, increasing utilisation, and achieving long term sustainability. While further research is required to confirm our results in other settings and to examine whether collaborative learning can truly deliver improvements in these areas, our study suggests that collaborative learning in YFHS is a valuable tool for achieving high quality, youth-friendly services for all young people.

## Abbreviations

CLS: Collaborative learning session/s
SDC: Swiss Agency for Development and Cooperation
UNICEF: United Nations Children’s Fund
WHO: World Health Organisation
YFHC: Youth-friendly health centre
YFHS: Youth-friendly health services
